# New results with regard to the Flora bust controversy: radiocarbon dating suggests nineteenth century origin

**DOI:** 10.1038/s41598-021-85505-x

**Published:** 2021-04-15

**Authors:** Ina Reiche, Lucile Beck, Ingrid Caffy

**Affiliations:** 1 PCMTH Team, Institut de Recherche de Chimie Paris (IRCP) – Centre de Recherche et de Restauration des Musées de France (C2RMF) - UMR 8247 CNRS, PSL University, ENSCP, 14 quai François Mitterrand, 75001 Paris, France; 2 Rathgen Forschungslabor, Staatliche Museen zu Berlin-Stiftung Preußischer Kulturbesitz, Schloßstraße 1a, 14059 Berlin, Germany; 3 Laboratoire de Mesure du Carbone 14 (LMC14) - LSCE/IPSL, CEA-CNRS-UVSQ, Université Paris-Saclay, Bât 450 porte 4E, 91191 Gif-sur-Yvette, France

**Keywords:** Soft materials, Biogeochemistry

## Abstract

Many works of art have been attributed to Leonardo da Vinci (1452–1519), the great artist-scientist-engineer of the Italian Renaissance; however, art historians have struggled to find definitive proof to connect Leonardo to these art pieces. The Flora wax bust in the Bode Museum, Berlin, was attributed to Leonardo because her face resembles several Leonardo portraits, but this attribution has the subject of intense debate since the bust’s acquisition in 1909. Using new chemical analyses and absolute ^14^C dating, we are able to resolve the question of authenticity. We show that the Flora wax bust is made primarily of spermaceti which was extracted from sperm whales. Therefore, ^14^C dating must consider the Marine Reservoir Effect. We have generated a new calibration method and dated the bust to the 19th c. This proves that the bust was not produced during the Renaissance, and thus cannot be attributed to da Vinci, and illustrates that ^14^C dating can be applied to unusual materials.

## Introduction

Radiocarbon (^14^C) dating has proved to be a valuable tool in cultural heritage research for determining the period in which a C-containing piece of artwork or archeological object was made. This method involves the carbon in cultural heritage materials in equilibrium with the ^14^C contained in the environment during the object’s genesis. A wealth of archaeological artifacts and pieces of art can be successfully dated thanks to the ^14^C dating method^1–4^ However, ^14^C dating is not always straightforward and prior analysis of the material to be dated is recommended. We report here on the chemical analysis and dating of the famous Flora wax bust (Inv. No. 5951) in the Bode Museum collection, National Museums in Berlin (Staatliche Museen zu Berlin, SMB—Stiftung Preußischer Kulturbesitz, SPK), which is attributed to Leonardo da Vinci because her face resembles the faces of figures in well-known Leonardo paintings.

## Historical background

**Figure 1:**
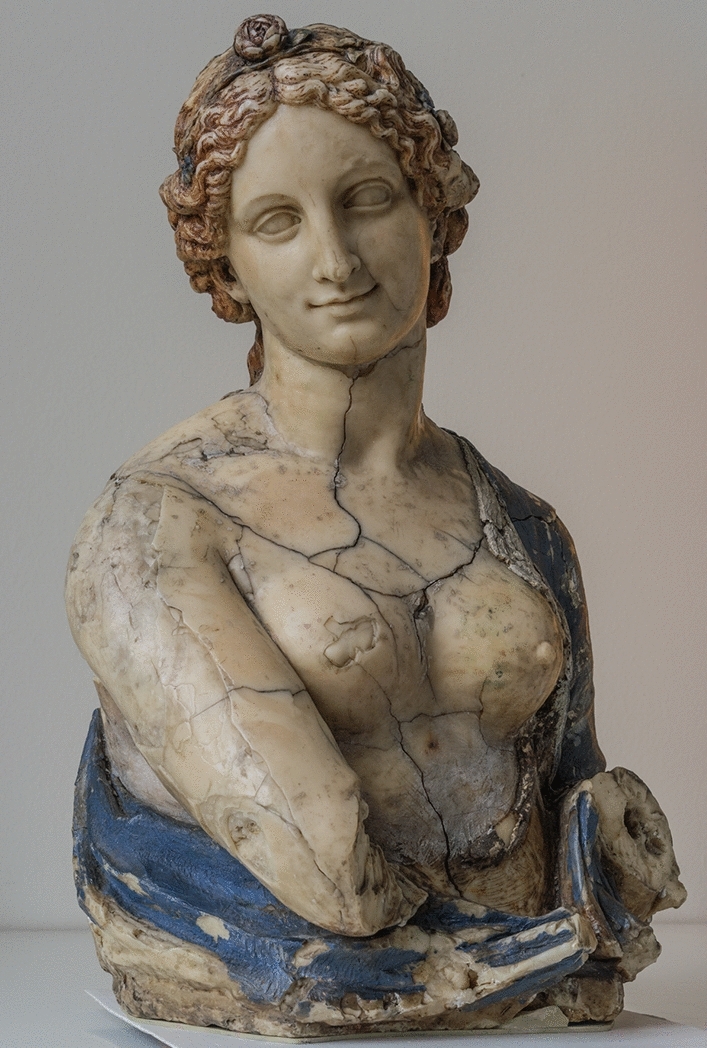
Flora bust, about 70 cm high, weight approx. 28 kg, Inv. No. 5951, Skulpturensammlung Museum für Byzantinische Kunst (SBM), Staatliche Museen zu Berlin (SMB), Stiftung Preußischer Kulturbesitz (SPK).

The attribution of the Flora bust has been the object of intense, long-lasting discussion since its acquisition in 1909 by the director general Wilhelm Bode for the Berlin Royal museums (Fig. 1 and S1). Two years after the acquisition, more than 730 articles were published in the German and English press as well as in France, Italy, Austria and Denmark arguing for and against the da Vinci attribution.

The controversy stems from the fact that the bust is made out of wax, an unusual material for an artwork in the Renaissance period. No other wax sculptures from this period is known yet Wilhelm Bode attributed the bust to Leonardo da Vinci upon acquisition. Other scholars such as Gustav Pauli (1866–1938), museum director in Hamburg, attribute it to Richard Cockle Lucas (1800–1883), a British 19th c. sculptor known to have created a large number of wax sculptures after ancient models^5^. Wilhelm Bode bought the bust in an auction in London for a high price (185,000 Goldmark) hoping to enlarge the Berlin collection with outstanding Renaissance objects. Immediately after the acquisition, analyses of photographs and the composition of the bust were carried out in an attempt to support Bodes attribution (Fig. S1).

## Previous research

In arguments against the bust’s production in the Renaissance, and instead that the bust was produced in the 19th c., researchers have relied on chemical analyses and historical documents. Chemists have observed the presence of spermaceti in the bust’s wax^6^, which was a rare material during Renaissance but very common in the 19th c. when it was used for candle wax and to create sculptures from 2D models^7^. In the 1980s a ^14^C dated sample indicated that the bust was not made in the Renaissance^8^. There are also historical documents that support a 19th c. attribution including a statutory declaration by the son of Richard Cockle Lucas stating that his father made the Flora bust in 1846 and a watercolour painting of the bust by Lucas’ son^5,8^. Additionally, evaluations of the casting method suggest that the bust could not have been produced during the Renaissance^8^. When the backside of the bust was opened it was found to contain a wood fragment, newspaper, and other materials from 19th c. It is however, possible that the objects could have been added later, when modifications were carried out. Lastly, it should be noted that there is no other known wax model from the Renaissance period.

The arguments for the bust’s production during the Renaissance, and thus attribution to Leonardo da Vinci, also use both chemical and stylistic evidence. Analyses in the 1900s of the wax proved inconclusive^9^, however an expert analysing the surface of the bust argued that the observed cracks were indicative of significant aging (Fig. S1)^10^. From a stylistic standpoint, experts have suggested that the polychromy was applied using techniques from the Renaissance, and that the Flora’s face closely resembles Leonardo’s other figures^11,12^. Additionally, researchers point out that while spermaceti wax was rare and expensive, it was in use during the Renaissance.Although the quality of the analyses was very high, the art-historical interpretation of the bust was likely influenced by the attribution that the researchers wished to be obtained, an attribution to Da Vinci (S1).

## Wax composition and radiocarbon dating

Wax used for artworks could be of animal origin (Chinese wax, lanolin, beeswax or spermaceti wax), vegetal origin (carnauba, ouricuri, candelila, esparto or Japan wax) or fossil origin (paraffin from petroleum). Beeswax is the most common wax, with documented use by early humans^13^. A corroded 6000-year-old small amulet discovered in Mehrgarh (Baluchistan, Pakistan) proves that beeswax has been used for lost wax casting since 4000 BC^14^. Spermaceti wax, which comes from the head cavity of the sperm whale, was identified in the Flora bust by chemical analysis in 1910^6^ and the 1980s^5^, and was commonly used in the 19th c. for cosmetics and candles. Various substances were commonly added to the Spermaceti wax mixture in order to modify the properties of wax. Typical additives include: tallow (animal fat), which increases the malleability and softness of the wax, and resins such as turpentine (composed of abietadienic acids, pimaradienic acids and cis-abienol or epimanool, larixol, larixyl acetate), which harden the wax. Pigments and dyes were also added and starch was used as an extender. Additionally, stearin, a mixture of palmitic and stearic acids, obtained from animal fats, has been used since 1831. Terpenoids of plant origin are also found in wax. Determining the composition of the wax is complicated because the wax, and the additives, might be altered through degradation processes. Only the wax markers that are stable over time should be considered for the identification of the wax type.

The bust could not be dated using ^14^C dating immediately after the acquisition in 1909 since ^14^C analysis was only developed by Willard Libby in 1946 shortly after the discovery of the radioisotope^15^. The earliest ^14^C dating of the Flora bust by Jürgen Freundlich dates from the 1980s^5^ and excludes the Renaissance period^5,8^. Freundlich used 3.2 g wax for ^14^C dating from below the left arm of the Flora sculpture. Conventional age determined was 290 ± 40 B.P. (KN-3224)^5^. As we will explain later a marine reservoir correction of the ^14^C dates was necessary and Freundlich provided a calendar date within the nineteenth century.

We think that we are able to resolve the mystery of the Flora bust dating through a series of comparative chemical analyses and absolute Accelerator Mass Spectrometry (AMS) ^14^C dating of well-sampled materials of the Flora bust as well as of two well dated 19th c. wax objects. Our study is based on observations made by X-ray radio/tomography and neutron tomography as well as endoscopy. The bust is hollow casted with at least three wax layers using the lost-wax molding. The most inner layer is the opaquest (pers. comm. Paul Hofmann, SBM, SMB-SPK).

## Present research

Two series of samples were studied, given that sampling must be limited on such a precious art object (Tables 1, S2). First, two small loose fragments (Flo-W1 and -W6, Fig. S2) and three small samples (F1–3, Fig. S3) on the surface of the bust were taken for the study of the chemical composition of the wax and its dating. In order to confirm the dating further samples were taken in a second series from the inside of the bust (Flora 1–4, Fig. S4), in order to exclude the possibility that the samples taken from the surface do not correspond to the original state of the bust. Additionally, the materials from the filling at the backside have also been sampled for dating (Flora 5, 6, 10, Fig. S4).

**Table 1:** Samples used for chemical analysis and AMS ^14^C dating.

Sample number	Material	PIXE	FT-IR	GC–MS	AMS ^14^C laboratory code SacA
Flo-W1^a^	Wax	x	x	x	54343^d^
	Lead white				54634^d^
	Wax				55025^d^
	Lead white				55026
Flo-W6^a^	Wax	x	–	x	54346
F1	Wax	–	x	x	54345
F2	Wax	–	–	x	54346
F3	Wax	–	–	x	54347
Flora 1	Undetermined	–	–	–	58288^e^
Flora 2	Blue wax	–	–	–	58289
Flora 3	Wax^c^	–	–	–	–
Flora 4	Mixture of waxes	–	–	–	58290^e^
Flora 5	Wooden spar (coniferous cut on half strand)^b^				58291
Flora 6	Textile	–	–	–	58292
Flora 10	Paper	–	–	–	58293
Leda-1	Wax	–	x	x	54348
Leda-2	Wax	–	x	x	54349
Leda 3	Wax	–	–	–	58295
Woman 1	Wax	–	–	–	58294

^a^Loose sample.

^b^Not enough material for dendrochronology (pers. comm. Catherine Lavier, dendrochronologist, C2RMF, Paris).

^c^Wax from external parts, sample too small to be dated.

^d^These samples were used for trials.

^e14^C dates were not calibrated since the composition was not known or uncertain.

Investigations were also performed on samples (Leda 1–3, Woman 1) from two bas-reliefs made by Richard Cockle Lucas (S3). The reliefs show two antique representations: “Leda and the swan” created in 1850 (22 cm × 17.8 cm × 3 cm, Alte Nationalgalerie, SMB-SPK, Inv.No. B II 433), S3: Fig. S5) and “Woman and winged woman” dated in 1848 (7,5 cm × 13 cm, SBM, SMB-SPK Inv. No. Lfd. Nr. 247).

For the characterisation of mineral wax constituents, 3 MeV microProton Induced X-ray Emission (microPIXE) at the NewAGLAE facility (C2RMF) was used^16,17^. Fourier transform infrared spectroscopy (FT-IR) and gas chromatography combined with mass spectrometry (GC–MS), at the Rathgen-Forschungslabor (Rathgen research laboratory), SMB-SPK were used for the determination of the wax composition, terrestrial or marine origin, which was necessary for calibrating the AMS ^14^C dating results. Radiocarbon dating of the Flora bust and two reliefs of Lucas: “Leda and the Swan” and “Woman and winged woman” were performed by AMS at the ARTEMIS facility (LMC14-LSCE)^18,19^.

## Results

### Wax chemical composition

**Figure 2:**
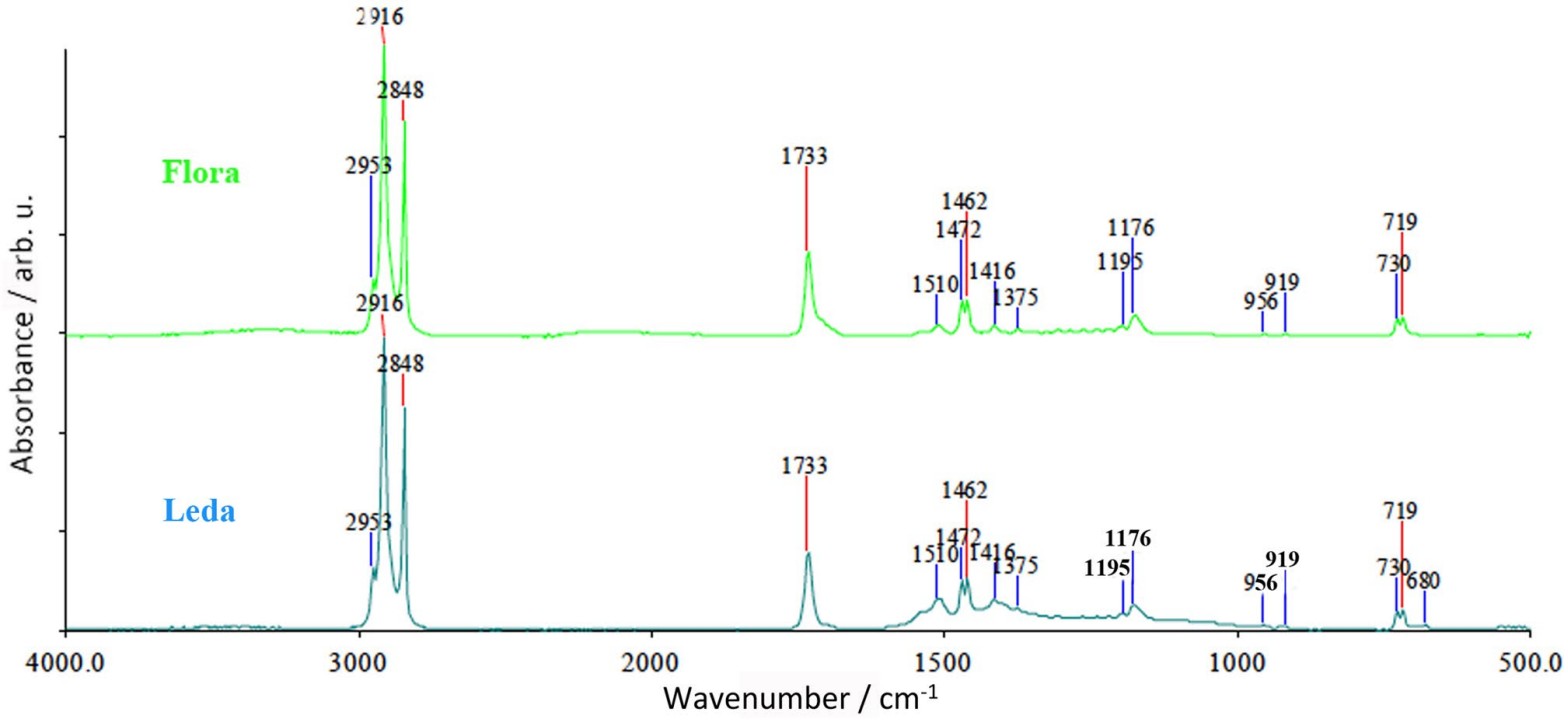
FT-IR spectra of the sample F1 of the Flora bust (above) compared to that of the sample Leda 1 (below).

FT-IR measurements allowed for the identification of wax constituents accompanied by lead stearates in the Flora bust and lead white in the Leda object. Beeswax and spermaceti are difficult to distinguish by FT-IR. The spectra of the samples of the Flora bust and the Leda relief (Fig. 2) showed very similar wax features (almost identical spectra). The spectra of both objects showed long chain aliphatic ν C–H bonding bands were found in the range of 2953–2848 cm^−1^, the sharp ester ν C=O band found at 1737 cm^−1^. Further small sharp bands can be attributed to in plane bonding of hydrogens of the phenyl group (between 1225 and 950 cm^−1^). The typical wax double bands are also observed at 730 and 719 cm^−1^ as well as the sharp bands at 1462 and 1375 cm^−1^. The remaining small sharp bands at 1510 and 1416 cm^−1^ can be attributed to v C=O bands of lead stearate and lead hydroxycarbonate. Lead white was found untransformed in wax samples from the Leda object made by Lucas. Lead stearate, which was found in both objects, could be a degradation product of lead white.

More detailed characterisation of the complex organic mixtures in the waxes was obtained by GC–MS. Stable biomarkers were identified by their different characteristic chromatographic patterns, which allows us to infer the original chemical composition.

Beeswax is composed of a homologous series of n-alkanes with odd carbon number, from C23–C33, n-heptacosane (C27) being the main compound. It also contains even numbered free fatty acids (C22–C34) and long-chain esters derived from palmitic acid ranging from C40 to C52. Because aging by partial sublimation and hydrolysis might lead to a change in the relative amounts of alkanes and esters, long chain alkanes and relatively stable esters are characteristic biomarkers of beeswax^13,20^.

Spermaceti wax is composed of even numbered long chain esters from C26 to C36 together with other esters in very low amount with odd carbon number in the range of C27–C33. Esters are mainly made of hexadecanoyl moieties associated with a fatty acid containing 10–18 carbon atoms. The degradation processes of spermaceti wax are not well known but because esters are known to be stable, the ester composition is most likely indicative of a spermaceti wax type.

**Figure 3:**
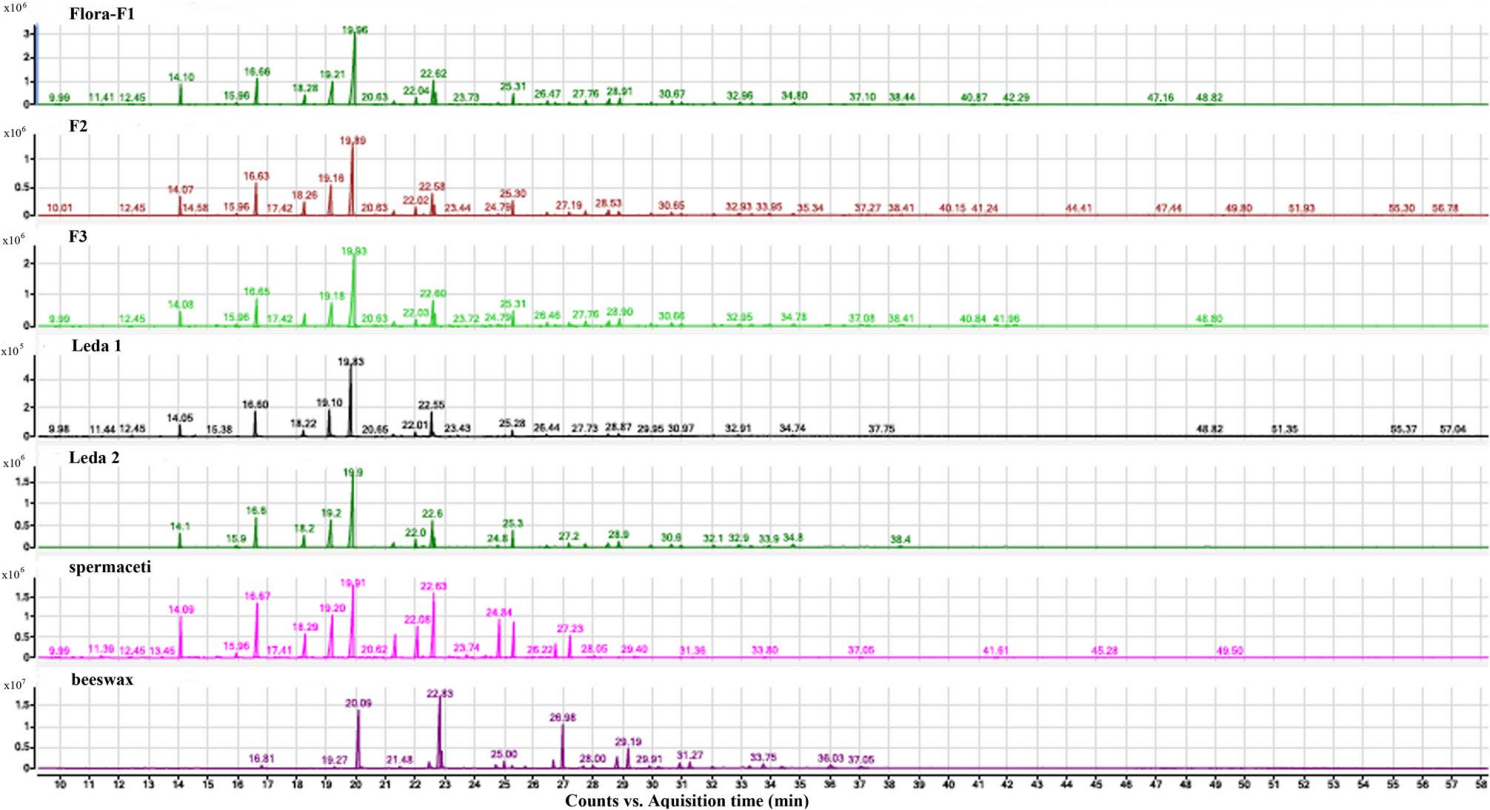
Chromatograms obtained after solvent extraction and derivatisation with MethPrepII (0.2 mol/L solution of m-tri-fluoromethylphenyl-trimethylammonium hydroxide in methanol) of the samples (from top to down): Flora F1–3, Leda 1–2 and the references spermaceti and beeswax.

GC–MS showed mixtures of various components, including long-chain hydrocarbons (alkanes), long-chain wax esters, long-chain alcohols and long-chain fatty acids in the Flora bust and in the Leda relief. All chromatograms of the Flora and the Leda samples show typical components for spermaceti type A, of sperm whale or cachalot (*Physeter Macrocephalus* L.) (Fig. 3)^21^. The main fatty acid ester components in the wax samples are tetradecyl tetradecanoate (C_30_H_60_O_2_) and hexadecyl hexadecanoate (C_32_H_64_O_2_) and hexadecyl dodecanoate (C_28_H_56_O_2_). There are also minor components representative of beeswax (*Apis mellifera*) specifically, a homologous series of the identified alkanes and typical saturated fatty acids. A high acid content and stearic acid are detected. Palmitic and stearic acids are commonly found in many natural binding media, such as fats or oils such as bees wax but their relatively high amount in the Flora bust lend us to conclude on the presence of stearin or possibly tallow in the wax mixture.

The results of the chemical characterization of the wax of the Flora bust and the “Leda and the swan” relief made by Richard Cockle Lucas in 1850 show that both compositions are very similar and principally composed of spermaceti with minor amounts of beeswax.

### Radiocarbon dating

Uncalibrated ^14^C results for materials derived from terrestrial organisms (wood, paper, textile) of the Flora bust are shown in Table 2 and for wax samples in Table 3. Uncalibrated ^14^C results obtained for two wax reliefs of Lucas: “Leda and the Swan” and “Woman and winged woman” are also given in Table 3. The ^14^C dates obtained for the wood, newspaper and textile fragments range from 110 ± 30 to 195 ± 30 BP with a combined uncalibrated date of 155 ± 20 BP. For the wax samples, ^14^C dates range from 340 ± 30 to 395 ± 30 BP for the Flora bust, from 380 ± 30 to 420 ± 30 BP for the “Leda and the Swan” relief and 380 ± 30 BP for the “Woman and winged woman” relief.

**Table 2:** Uncalibrated and calibrated ^14^C dates of the wood, textile and paper samples of the Flora bust.

AMS ^14^C dating laboratory code SacA	Sample reference	Uncalibrated ^14^C age BP (years)	Calibrated dates (years AD) (95.4%)
58291	Flora 5 wood	110 ± 30	1682–1938
58292	Flora 6 textile	195 ± 30	1646–1950
58293	Flora 10 paper	170 ± 30	1656–1950
Combination	Flora bust-terrestrial materials	155 ± 20	1667–1950

**Table 3:** Uncalibrated and calibrated ^14^C dates for the wax samples of the Flora bust and for two wax reliefs of Lucas: “Leda and the Swan” and “Woman and winged woman”.

AMS ^14^C laboratory code SacA	Artwork and expected date of creation in ()	Sample reference	Uncalibrated ^14^C age BP (years)	Calibrated ^14^C dates using a combination of 15% atmospheric/85% marine (± 10%) calibration curves (years AD)
54343	Leda and the Swan (1850)	Leda 1	415 ± 30	1704–1950
54344		Leda 2	420 ± 30	1704–1950
58295		Leda 3	380 ± 30	1705–1950
58294	Woman and winged woman (1848)	Woman 1	380 ± 30	1705–1950
58289	Flora bust (unknown)	Flora 2	350 ± 30	1710–1950
54347		Flora F1	370 ± 30	1706–1950
54348		Flora F2	385 ± 30	1704–1950
54349		Flora F3	385 ± 30	1704–1950
55026		Flora W1	340 ± 30	1713–1950
54346		Flora W6	395 ± 30	1704–1950

## Discussion

**Figure 4:**
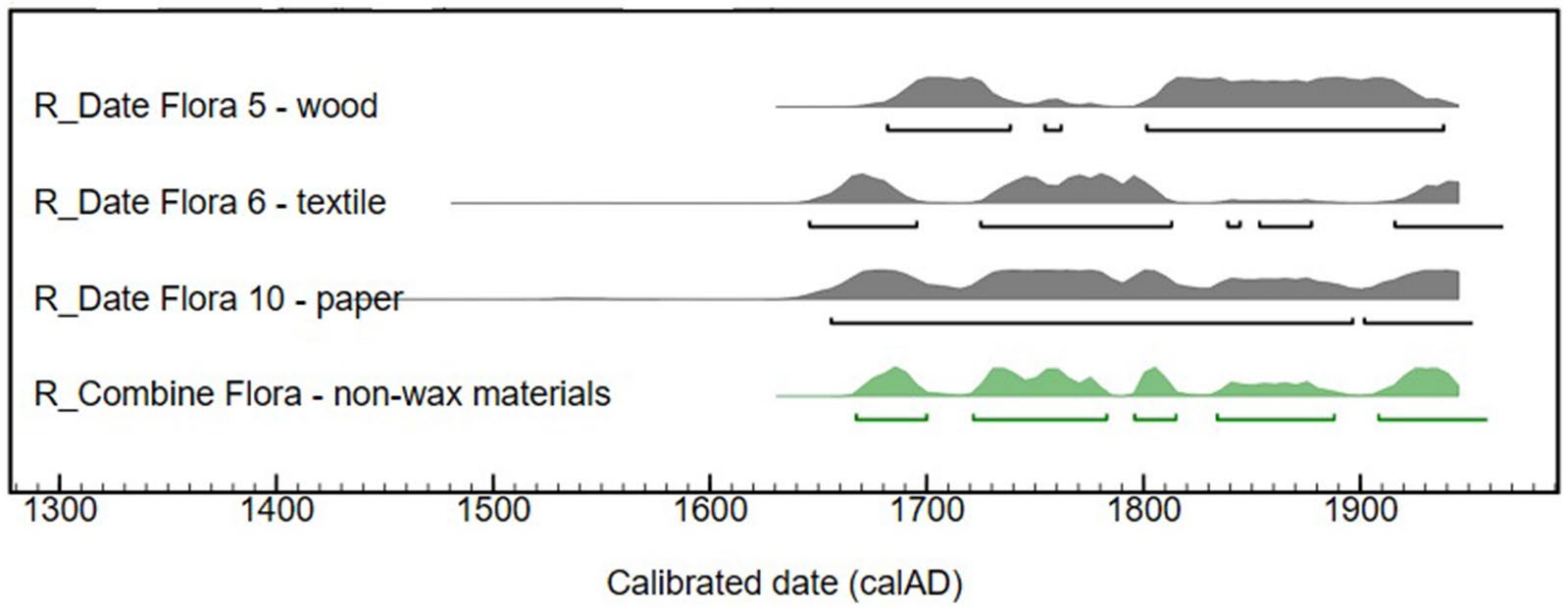
Calibrated ^14^C dates for wood, textile and paper samples taken from the Flora bust (in grey). The statistical combination of the three dates in green gives the interval of 1667–1950 AD. The χ^2^ test value of T = 4.1 (5% 6.0) shows their consistency.

Based on the composition of the dated samples, two calibration procedures must be undertaken to transform the radiocarbon (^14^C) dates into accurate calendar dates. The ^14^C dates of the wood, newspaper and textile fragments were calibrated using the IntCal20 atmospheric calibration curve^22^ (Table 2, Fig. 4). All results are statistically consistent and give calibrated dates between 1646 and 1950 AD. The combination of the three dates provides the interval 1667–1950 AD. The elongated distribution is due to the flat shape of the calibration curve for this period^23^. Nevertheless, the results show that all the wood, newspaper and textile samples found inside the statue definitively date after 1650.

To calibrate the ^14^C dates obtained from the wax samples, the composition of the material has to be carefully considered. The Flora bust and “Leda and the swan” relief waxes are principally composed of spermaceti from a sperm whale that lives in the ocean, mixed with minor amounts of beeswax and other organic compounds extracted from terrestrial animals. The wax is thus primarily composed of marine material with some of terrestrial origin. The ^14^C source of terrestrial animals is in equilibrium with the atmosphere whereas that of whales ^14^C source is subject to the Marine Reservoir Effect (MRE)^24^. The MRE affects ^14^C dates since carbon consumed by organisms in the ocean is older than that consumed on land. Because the wax used for the sculptures is composed of carbon from different sources, other than just atmospheric carbon, the ^14^C measurements produce apparent old uncalibrated radiocarbon ages from 340 to 420 BP (Table 3) and a correction is needed to compensate this effect in calibration calculations.

The mixture of marine and terrestrial sources in the wax requires the use of a combination of two calibration curves: IntCal20 atmospheric^22^ and Marine20 marine^25^, both weighted by the proportion of terrestrial and marine materials. In the case of the Flora bust, the determination of the exact ratio of spermaceti wax and terrestrial wax was not feasible because only a few samples of wax were available for analysis.

To further complicate the procedure, the location of the marine source must be known to accurately calibrate marine material. Whales travel long distances, integrating the reservoir ages of the different water masses along their paths making that the determination of the marine reservoir age (MRA) for whale material ^14^C dates difficult. The global-average (MRA) of surface waters is c. 500 years^25^ but values range from about 400 years in subtropical oceans to over 1000 years in the poles. According to our knowledge no MRA has been reported for sperm whale (*Physeter Macrocephalus* L.) bone or for spermaceti except the estimation of 300 ± 200 years made by Freundlich^5^. Various values can be found for other cetacean materials in literature. One of the more complete studies, which is based on the analysis of 21 whales caught in Norway during the 19th c., proposed an average marine reservoir age (MRA) of 370 ± 30 years for various whales from the North Atlantic^26^. Previous publications recommended to use a c. 200 years marine reservoir correction for bowhead whales from Canadian Artic^27^, or determined a mean value correction of 320 ± 35 years for marine mammals, including whales, living near Sweden^28^ or c. 350 years correction for a 17th c. Finnback whale bone collected in Spitsbergen^29^. Additionally, based on an exhaustive compilation of published marine mammal radiocarbon dates, both live-harvested materials and subfossils, from the Canadian Arctic Archipelago, Furze et al.^30^ provided reservoir offset values for beluga (*D. leucas*) and bowhead (*B. mysticetus*) corresponding to a MRA of 570 ± 95 years for the latter.

### Calibration of the ^14^C dates of the 19th c. wax objects made by Richard Cockle Lucas

**Figure 5:**
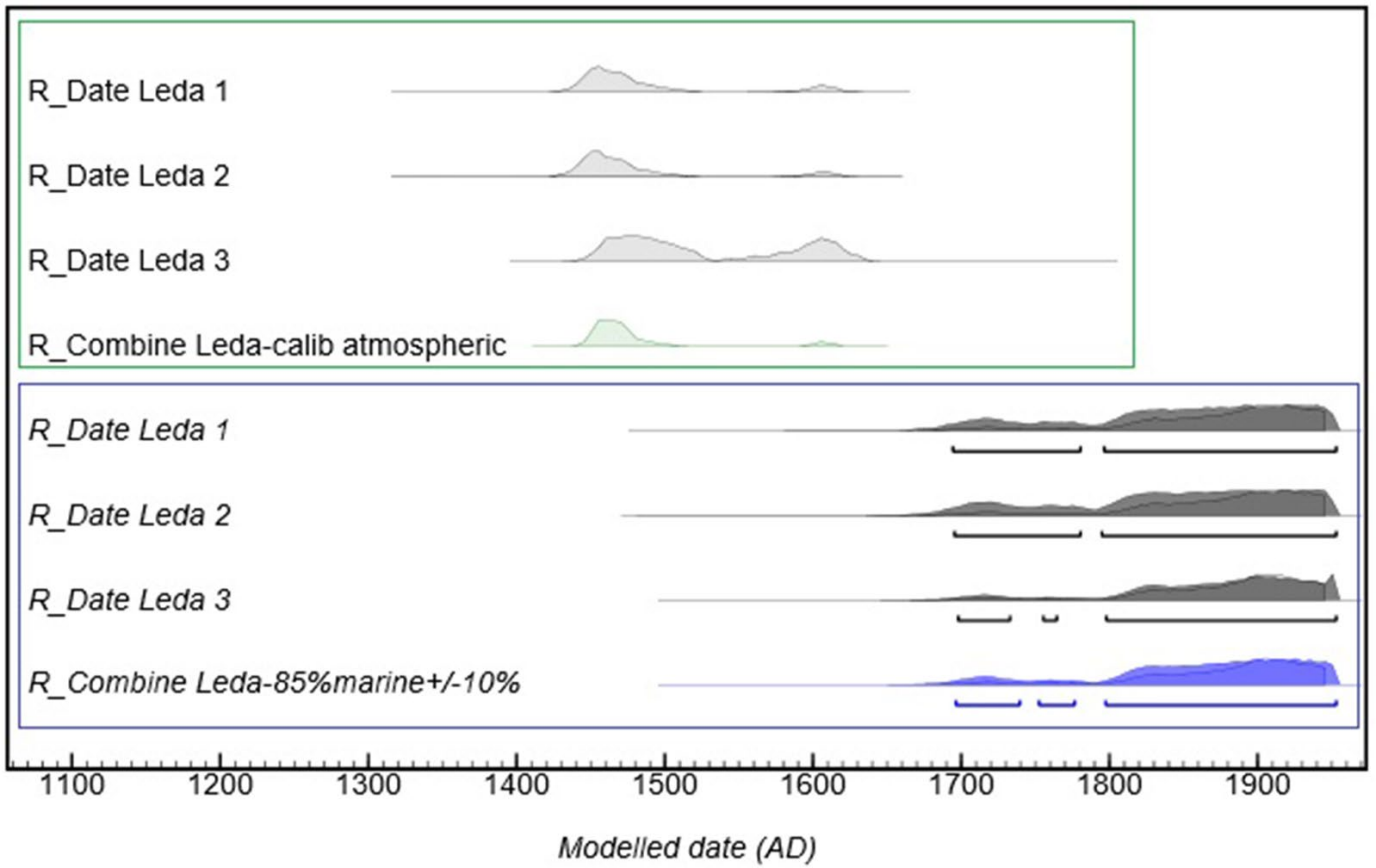
Calibrated ^14^C dates for the wax samples of the Leda and the Swan relief using atmospheric curve only, in light grey and light green, give dates out of range of the known date of creation of this artwork made by Lucas in 1850. A calibration of the same samples with a combination of 15% atmospheric/85% marine (± 10%) calibration curves, in dark grey, gives dates in the time frame of the relief’s creation in 1850. The statistical combination of the three dates, in blue, gives the interval of 1704–1950 AD.

Since the spermaceti MRA value and the spermaceti wax content cannot be determined precisely, another approach was developed to calibrate the ^14^C dates of the Flora bust. This approach is based on the well-dated wax relief, “Leda and the Swan”. This relief was created by R. C. Lucas in 1850 and the chemical analysis has shown that its composition is similar to that of the Flora bust (Figs. 2, 3). The “Leda and the Swan” relief was used as reference to determine the appropriate combination of the IntCal20 and Marine20 calibration curves to be applied to the Flora wax material. The percentage of each curve was established by adjusting the calibrated date distribution of the Leda relief on both sides of the year 1850. To obtain this result, a combination of 15% atmospheric/85% marine curves was selected with an uncertainty of 10% to reflect material variability. The resulting distribution of dates is from 1704 to 1950 AD (Table 3, lower part of Fig. 5) which is not very precise, but this method has the advantage to take into account uncertainties on spermaceti MRA and on the spermaceti/beeswax content ratio. Figure 5 also shows that the results calibrated with the IntCal20 atmospheric curve are inconsistent with the known date of creation of the “Leda and the Swan” relief, which confirms the presence of marine material in the wax.

### Calibration of the ^14^C dates of the Flora bust

**Figure 6:**
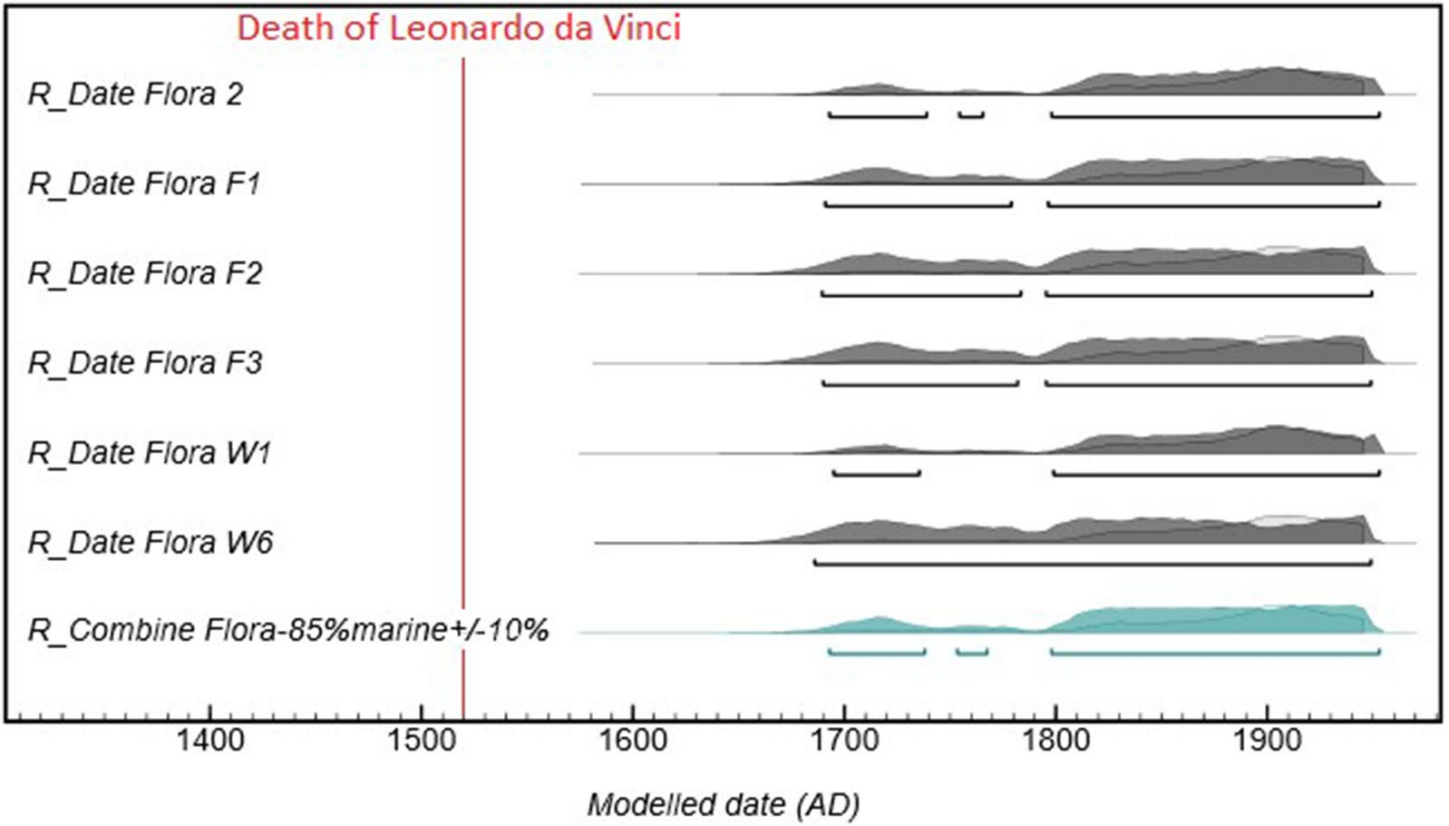
Calibrated ^14^C dates for the wax samples of the Flora bust using a combination of 15% atmospheric/85% marine (± 10%) calibration curves (in dark grey). The statistical combination of the three dates in blue gives the interval of 1707–1950 AD.

The same combination of atmospheric and marine calibration curves was applied to calibrate the ^14^C dates obtained for wax samples taken from six different locations at the surface and inside of the Flora bust because the composition of the Flora is similar to that of the Lucas wax objects. The results are presented in Fig. 6 and Table 3. All the dates are after 1704 AD, with a statistical combination on the six dates of 1712–1950. Uncertainty on the calibration curves lead to a broad interval for the dates of the Flora wax with about two centuries precision. Calibrated dates obtained on the wax samples, when the MRE is taken into account, agree with those of the wood, paper and textile samples, which confirms the strength and validity of our approach. All of the analysed constituents of the Flora bust are dated after 1700 AD, precluding the bust from being created in the Renaissance period.

Chemical analyses and absolute dating were performed on different materials and several wax samples taken from the surface and inner parts from the Flora bust as well as on two dated wax reliefs made by the British 19th c. sculptor Richard Cockle Lucas, who some claim is the author of the Flora bust. The Lucas object “Leda and the swan” dated at 1850 could only be accurately dated using ^14^C measurements when a mixed terrestrial and marine calibration was taken into consideration because the wax is primarily made from spermaceti with minor amount of beeswax. Because the spermaceti was extracted from sperm whales living in deep and shallow seawaters, ^14^C dating must to consider the MRE. The Flora bust was shown to have an extremely similar composition to the Lucas object. Thus the same calibration correction procedure was applied to the uncalibrated ^14^C dates of the Flora bust. This new procedure involved calibrating of the ^14^C dates by considering a combination of 85% marine/15% atmospheric curves. The result dates the Flora materials to the 18-19th c., which proves that the bust was not produced during the Renaissance, and therefore cannot be attributed to Leonardo. This study also illustrates that ^14^C dating must take into account the heterogeneity and diversity of art objects, some of which may contain uncommon materials such as spermaceti wax.

While it is somewhat disappointing to learn that the bust cannot be attributed to Leonardo, this information does provide useful insight into history. The sperm whale population suffered a serious decline in the 1740s when sperm whaling started on an industrial scale. The use of spermaceti in art objects shows how widespread the use of sperm whale products was and highlights the whaling industry’s importance during the industrial revolution. Other culturally significant objects may also be composed of materials that show the importance of certain industries or materials. There is clearly a need for art historical research to integrate natural science investigations in order to provide information allowing an improved attribution of art works and allowing to give another dimension to the historical value of such objects.

## Methods

Considering the precious nature of the wax bust and reliefs no statistical methods could be used to predetermine sample location and size. The experiments were not randomized and investigators were not blinded to allocation during experiments and outcome assessment.

### Micro-proton induced X-ray emission (PIXE) analyses

Micro-PIXE analyses were conducted at the external micro-beam line of the 2 MV tandem particle accelerator NewAGLAE at the C2RMF in Paris. The proton beam (3-meV, ca. 50 µm in diameter) was directed at the samples under atmospheric pressure with helium purging. Major, minor, and trace elements from Na to Pb were measured using four X-ray SDD detectors by PIXE^17^. Results are shown in the Supplementary Information: S4, Fig. S6.

### Fourier transform infrared spectroscopy (FT-IR)

Fourier transform infrared spectroscopy (FT-IR) was performed by a Paragon 1000 PC type FT-IR spectrometer coupled with an FT-IR microscope from Perkin Elmer in transmission in the range of 4000–500 cm^−1^. The spectral resolution is 4 cm^−1^. The samples were prepared on a diamond measuring cell from High Pressure Diamond Optics. The FTIR spectra obtained from the samples are compared with reference spectra from the self-built RF, the IRUG and Sadtler databases. The origin of individual spectral bands is interpreted^31^.

### Gas chromatography combined with mass spectrometry (GC–MS)

GC–MS analyses were carried out with a GC/MSD system from Agilent (GC 7890B, MSD 5977A). The samples (Table 4) were extracted with various solvents (isooctane or methanol) and then derivatized. Two derivatizing agents were used, MethPrepII (0.2 mol/L solution of m-trifluoromethylphenyl trimethylammonium hydroxide in methanol) and BSTFA (N, O-bis (trimethylsilyl) trifluoroacet-amide). 1 μL of the respective sample solution (isooctane extract; dissolved derivatized sample) was injected (splitless) into the injector heated to 300 °C using an autosampler and transported using a helium flow of 1.2 mL/min. The chromatographic separation was carried out on a DB-5MS column, with an internal diameter of 0.25 mm, a film thickness of 0.25 μm and a length of 30 m. The following GC oven temperature program was used: start temperature 80 °C (3 min isothermal) first temperature gradient from 10 °C/min to 200 °C (3 min isothermal), second temperature gradient from 20 °C /min to 300 °C (30 min isothermal). The interface temperature between GC and MS was 300 °C. The mass fragmentation of the individual molecules was carried out by means of an electron impact excitation at 70 eV. The MS detector ran at an ion source temperature of 200 °C and an MS quadrupole temperature of 150 °C. The following scan segment was used: from 9.30 min 40–600 amu. The mass spectra were evaluated using the NIST database (version 2.2), the evaluation software AMDIS, the ESCAPE database and Regert et al.^21^. The areas of the peaks in the total ion chromatogram served as a reference point for an approximate quantitative comparison between the chromatograms of the different samples. Before the analysis, however, no standards of pure components were measured. Thus, only semi-quantitative results can be achieved, which are classified as sufficient for solving the identification. A spermaceti reference sample, ten beeswax samples and carnauba and paraffin wax samples from the Rathgen research laboratory were measured in order to obtain typical compounds and the retention times of these compounds for the method used and to be able to carry out corresponding comparisons^31^.

**Table 4:** Overview of the samples analyzed by GC–MS, GC–MS method and information on sample preparation (extraction or derivatization) and GC–MS file name.

Sample description	GC–MS method	File name	Remarks
Flo-W1_Isooct	1806_Binder60min_splitless.M	180608_01 Flo-W1_Isooct_BM60minsplitless	Flo-W1, Isooctane extract
Flo-W6_Isooct	1806_Binder60min_splitless.M	180608_02 Flo-W6_Isooct_BM60minsplitless	Flo-W6, Isooctane extract
Flo-W1_Isooct_ BSTFA	1806_Binder60min_splitless.M	180608_03 Flo-W1_Isooct_BSTFA_BM60min splitless	Flo-W1, Isooctane extract, BSTFA derivatization
Flo-W6_Isooct_ BSTFA	1806_Binder60min_splitless.M	180608_04 Flo-W6_Isooct_BSTFA_BM60min splitless	Flo-W6, Isooctane extract, BSTFA derivatization
Flo-W1_Isooct_ BSTFA_1h	1806_Binder60min_splitless.M	180608_07 Flo-W1_Isooct_BSTFA_1h_BM60min splitless	Flo-W1, Isooctane extract BSTFA Derivatization (1 h)
Flo-W6_Isooct_ BSTFA_1h	1806_Binder60min_splitless.M	180608_08 Flo-W6_Isooct_BSTFA_1h_BM60min splitless	Flo-W6, Isooctane extract, BSTFA Derivatization (1 h)
FloW1_MPII	1806_Binder60min_splitlessMP.M	180614_10 FloW1_MPII_BM60minsplitlessMP	Flo-W1, MethPrepII Derivatization
FloW6_MPII	1806_Binder60min_splitlessMP.M	180614_11 FloW6_MPII_BM60minsplitlessMP	Flora-W6, MethPrepII Derivatization
FloP1_MPII	1806_Binder60min_splitlessMP.M	180614_12 FloP1_MPII_BM60minsplitlessMP	F1, MethPrepII Derivatization
FloP2_MPII	1806_Binder60min_splitlessMP.M	180614_13 FloP2_MPII_BM60minsplitlessMP	F2, MethPrepII Derivatization
FloP3_MPII	1806_Binder60min_splitlessMP.M	180614_14 FloP3_MPII_BM60minsplitlessMP	F3, MethPrepII Derivatization
LedaP1_MPII	1806_Binder60min_splitlessMP.M	180614_16 LedaP1_MPII_BM60minsplitlessMP	Leda-1, MethPrepII Derivatization
LedaP2_MPII	1806_Binder60min_splitlessMP.M	180614_22 LedaP2_MPII_BM60minsplitlessMP	Leda-2, MethPrepII Derivatization

### AMS ^14^C dating

For AMS ^14^C dating^32,18^, between 1 and 5 mg of material were collected. All the samples (except W1) were prepared according to the classical procedure for organic materials^18^. The textile and paper samples were pretreated with acid (0.5 M HCl at 80 °C for 1 h) and the wax samples were cleaned mechanically. Samples were dried under vacuum overnight (60 °C under 0.1 mbar) and then placed in quartz tubes with excess of CuO (400–500 mg) and 1-cm Ag wire. The quartz tubes were sealed under vacuum (5 × 10^−6^ mbar) and heated at 850 °C for 5 h. Sample W1 was heated at 400 °C. CO_2_ gas was produced and separated from H_2_O using a dry-ice/alcohol trap (− 78 °C). CO_2_ samples were reduced to graphite targets by hydrogen over iron catalyst. Carbon isotopes were measured with the AMS LMC14/ARTEMIS facility (Saclay, France)^19^. The ^14^C dates were calibrated using the OxCal4.4 software^33^

## Supplementary Information

Supplementary Information.

## Data Availability

All relevant data are available from the Rathgen research laboratory under the report number RF 38_050718 Rathgen-Forschungslabor, Staatliche Museen zu Berlin, Stiftung Preußischer Kulturbesitz, Berlin, 2019^31^ and PCMTH IR 2020_03 Institut de Recherche de Chimie Paris—Centre de recherche et de restauration des musées de France, 2020^32^.
