# Supplementary material for: New results with regard to the Flora bust controversy: radiocarbon dating suggests nineteenth century origin
Source: Sci Rep. 2021 Apr 15;11:8249. doi: 10.1038/s41598-021-85505-x (PMC8050239; doi:10.1038/s41598-021-85505-x)
Supplement: Supplementary file 1 — Supplementary Information. [file 41598_2021_85505_MOESM1_ESM.pdf]

Supplementary information related to the manuscript:

New results with regard to the Flora bust controversy: radiocarbon dating suggests 19th century origin

Ina Reiche*[a, b], Lucile Beck[c], Ingrid Caffy [c]

[a] Dr. habil. I. Reiche *Corresponding Author(s)
present address: PCMTH team
PSL university, ENSCP, Institut de Recherche de Chimie Paris (IRCP) – Centre de recherche et de restauration des musées de France (C2RMF) - UMR 8247 CNRS
14 quai François Mitterrand, 75001 Paris, France
E-mail: ina.reiche@chimieparistech.psl.eu

[b] Dr. habil. I. Reiche
Rathgen Forschungslabor
Staatliche Museen zu Berlin-Stiftung Preußischer Kulturbesitz
14059 Berlin, Schloßstraße 1a, Germany

[c] Dr. Lucile Beck, Ingrid Caffy
Laboratoire de Mesure du Carbone 14 (LMC14), LSCE/IPSL, CEA-CNRS-UVSQ, Université Paris-Saclay
Bât 450 porte 4E, CEA Saclay, 91191 Gif-sur-Yvette, France

S1: Flora bust in 1909 and controversy

The arguments for the production during the Renaissance (and then possibly by the Leonardo da Vinci) are 1) that in 1909/10 Carl Brittner of the Chemical laboratory of the Royal Museums of Berlin, under the direction of Friedrich Rathgen, analysed the wax of the Flora bust and other works by Lucas by testing the fusion points. Fusion points were variable and so no date could be provided [1]. Thus, the attribution remained inconclusive, the bust could have been made by Leonardo or Lucas. 2) Adolf Miethe (1862-1927) [2], who has directed since 1899 the Photochemical Laboratory of the Royal Technical High school in Berlin-Charlottenburg, studied photographs in order to evaluate the preservation state of the bust (such as Fig. S1). He claimed that the cracks are indicative of an old age. 3) Prof. Dr. Eduard Raehlmann (1848-1917) looked at the polychromy and stated that it was created according to ancient paint techniques known in the Renaissance. Therefore, he concluded that the bust was made during the Renaissance[3]. 4) Josef Riederer supported the analysis of Raehlmann when he copied the statement by Raehlmann. But Riederer did not perform any of his own analyses [4]. 5) Spermaceti wax material was in use during the Renaissance, however, it was extremely rare and expensive. 6) The stylistic similarity of the figure's face with those of the Leonardo school and Leonardo was known for testing new, original material for his works.
Arguments against a production during the Renaissance, and then excluding the attribution to Leonardo and more likely to Lucas, are 1) that there is a statutory declaration by the son of Richard Cockle Lucas that his father made the Flora bust in 1846. The declaration also stated that the sculptor made the bust after a painting of Flora by a painter from the Bernardo Luini workshop, Leonardo School (kept in the Morrison Collection, Basildon Park) [5]. 2) There is a watercolour copy of the bust, which was made by the son of Richard Cockle Lucas (Albrecht Dürer Lucas), based on the bust stored in Lucas' house. This shows that the son has seen the bust in the house of his father, when it was in his possession [6]. 3) In 1910 Martin Schauss evaluated the bust and casting technique. Schauss stated that such a round wax cast could not be created in Leonardo's time. [6] 4) The chemist Georg Pinkus identified spermaceti as well as palmitic acid in the wax, both of which were not common during Renaissance but very common in the 19th c. [7,8] In the 19th c. spermaceti and palmitic acid were commonly used for candle wax and to create sculptures from 2D models[9]. 5) Further chemical analyses with Fourier Transform Infrared Spectroscopy (FT-IR), by Hermann Kühn of the Doerner Institute Munich, confirmed that the bust wax is composed of 90% spermaceti. A sample from the wax copy « Athena » of the Parthenon (a statuette on plinth, with figures in relief, 28 in. high, no. 13 of the catalogue "The remaining works of Lucas") which is definitively attributed to Lucas, showed a similar chemical composition than that of the Flora bust. 6) Jürgen Freundlich performed ^{14}C dating in

1984 considering the Marine reservoir effect (MRE) for the spermaceti containing wax and excluded the Renaissance theory [6]. 7) The backside of the bust was opened by Friedrich Rathgen. Inside a wood fragment, newspaper, and other materials from 19th c. were found. These objects could have been added later to the statue, when modifications were carried out most likely by Richard Cockle Lucas, who kept the statue in his house, but these findings should be taken into account. 8) There are no other known wax models from the Renaissance period.

References

1 Rathgen, F. Über die Untersuchung des Wachses der Florabüste. *Chemiker Zeitung* Jahrgang XXXIV (1910).

2 Miethe, A. & Raehlmann, D. E. Zur Frage der Florabüste im Kaiser-Friedrich-Museum. *Amtliche Berichte aus den Königlichen Kunstsammlungen* 31, 13–16 (1910).

3 Raehlmann, E., Bouet, E. & Bode, W. Zur Frage der Florabüste im Kaiser-Friedrich-Museum. *Amtliche Berichte aus den Königlichen Kunstsammlungen* 32, 10–13 (1911).

4 Riederer, J. *Kunstwerke chemisch betrachtet*. (Springer, 1981).

5 Wolff-Thomsen, U. *Die Wachsbüste einer Flora in der Berliner Skulpturensammlung und das System Wilhelm Bode. Leonardo da Vinci oder Richard Cockle Lucas?* , 278 (Verlag Ludwig, 2006).

6 Ost, H. *Falsche Frauen. Zur Flora im Berliner und zur Klytia im Britischen Museum*. (Verlag der Buchhandlung Walther König, 1984).

7 Pinkus, G. Wachs der Florabüste. *Chemiker Zeitung* XXXIV, 577f (1910).

8 Pinkus, G. *Chemiker Zeitung* XXXIV, 353f (1910).

9 Kühn, H. *Conservation and restoration of works of art and antiquities*. (Butterworths, 1986).

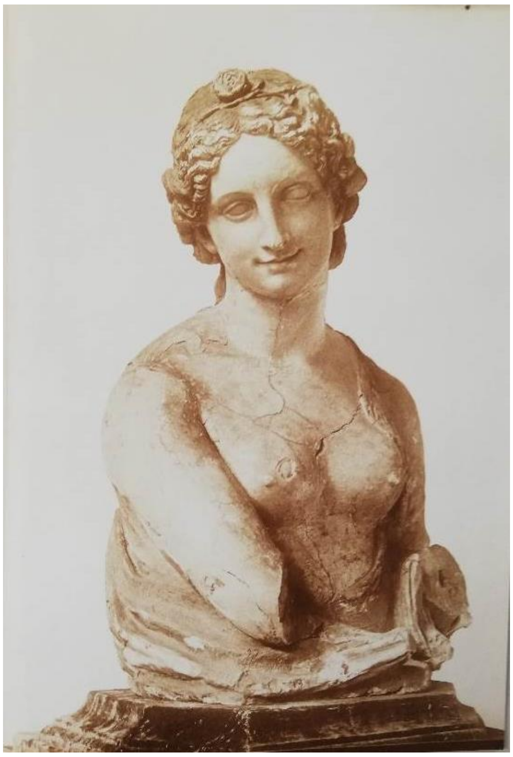

Figure S1. Archival picture of Flora bust from 1909 (Zentralarchiv, SMB-SPK, ZA I, SKS 176), about 70 cm high, weight approx. 28 kg, Inv. No. 5951, Skulpturensammlung Museum für Byzantinische Kunst (SBM), Staatliche Museen zu Berlin, Stiftung Preußischer Kulturbesitz (SMB-SPK).

S2: Description of the samples taken from the Flora bust and the two objects made by Richard Cockle Lucas

First series: The two loose samples (Flo-W1 and W6) are sections of the loose fragments W1 and W6 of the Flora bust indicated by the red arrows in the picture in Figure S2.

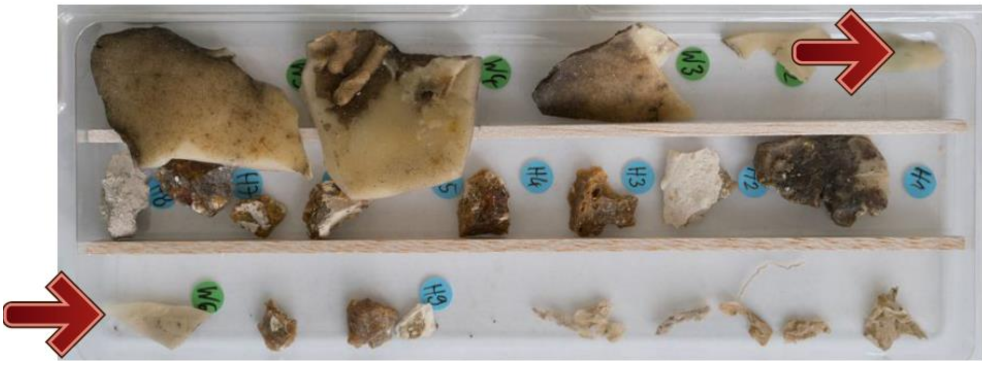

Figure S2. Picture of the loose samples of the Flora bust (Inv. No. 5951) kept in the collection of the Skulpturensammlung - Museum für Byzantinische Kunst (SBM), SMB-SPK.

Three samples (F1-3) were taken from the Flora bust at the locations indicated on Figure S3.

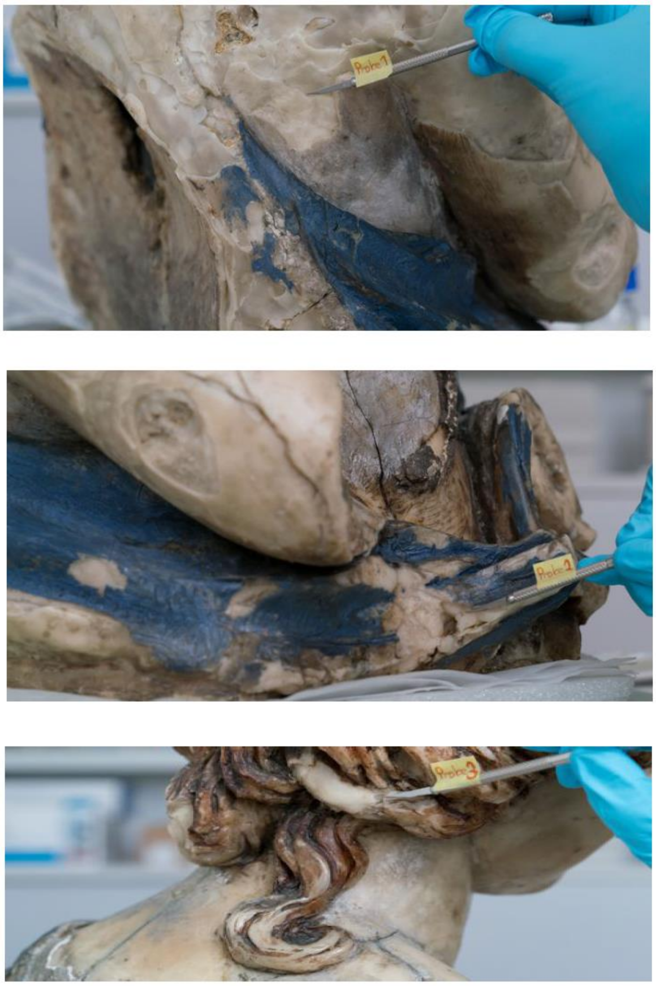

Figure S3. First series of samples (F1-3) of the Flora bust (Inv. No. 5951) kept in the collection of the Skulpturensammlung - Museum für Byzantinische Kunst (SBM), SMB-SPK.

Second series: Further samples of the wax and other materials from the inner parts (Flora 1-10) were taken from the Flora bust at the following locations (Figure S4).

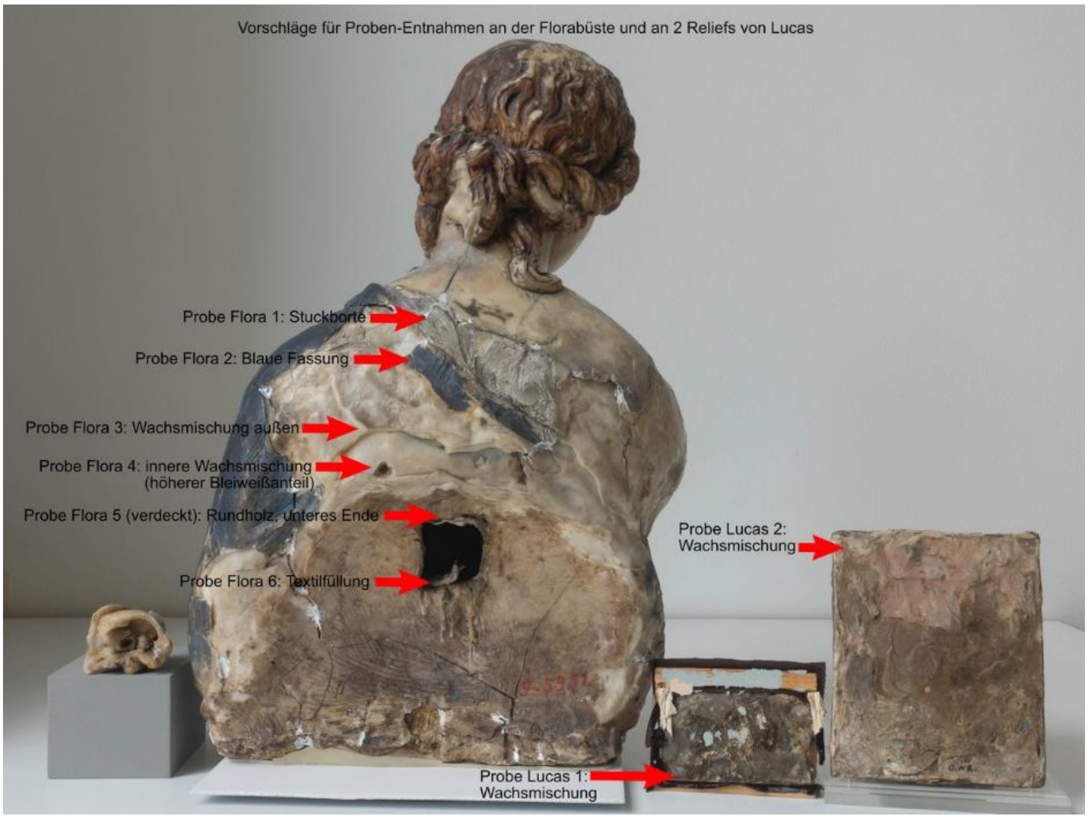

Figure S4. (from left to right) Arrows marking the second series of samples (Flora 1-10) of the Flora bust (Inv. No. 5951), center Lucas 1 “Woman and winged woman” (SBM Inv. No. Lfd. Nr. 247) and far right, Lucas 2 „Leda and the swan“ (ANG Inv. No. B II 433). All of which are kept in the collection of the National Museums in Berlin (SMB-SPK).

S3: Description of samples taken on the two objects made by Richard Cockle Lucas

A wax bas-relief, “Leda and the swan,” which is said to have been created by Richard Cockle Lucas in 1850 (1800-1883) (Alte Nationalgalerie, Inv. No. B II 433, 22 cm x 17.8 cm x 3 cm) was sampled. It is a scaled down copy of an ancient Greek stone relief that has been in the British Museum since 1813 (courtesy of Neville Rowley, Gemäldegalerie, SMB-SPK). Link to the original:
http://www.britishmuseum.org/research/collection_online/collection_object_details.aspx?objectId=406238&partId=1
Two samples at broken out places (Leda 1 and Leda 2) are indicated on the figure S5 with the red arrows.

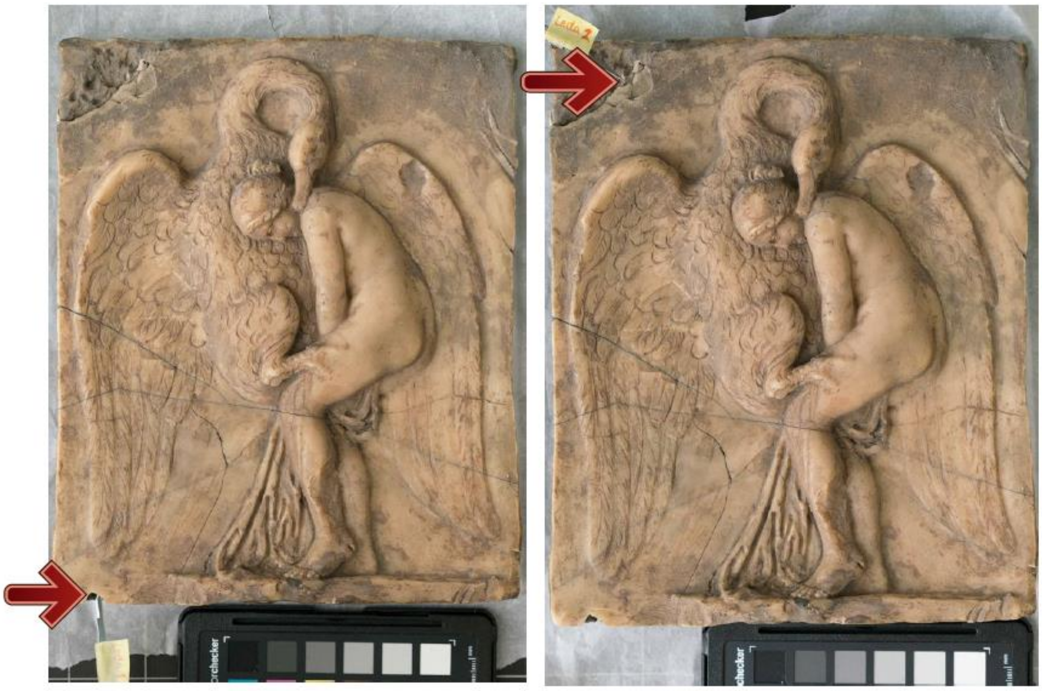

Figure S5. First series of samples (Leda 1 and 2) of the “Leda with a swan” wax relief (ANG Inv. No. B II 433).

Two further samples were taken in the second analysis series: another one from the “Leda and the swan” relief (Leda 3) and a second one (Lucas 1) from another dated wax object, a relief with an antique representation (“Woman head and a winged woman”, Bode-Museum Inv. No. Lfd. Nr. 247, 7,5 cm x 13 cm) made by Richard Cockle Lucas in 1848 (Figure S4).

S4: 3 MeV PIXE spectrum of the wax sample Flo-W1 indicating the traces of lead in the wax

Traces of lead were detected in the wax of the Flora bust by 3 MeV micro-PIXE. This indicates the presence of a lead-containing compound added to the wax (Figure S6), which could be lead white, a compound that is frequently added to wax in order to opacify it.

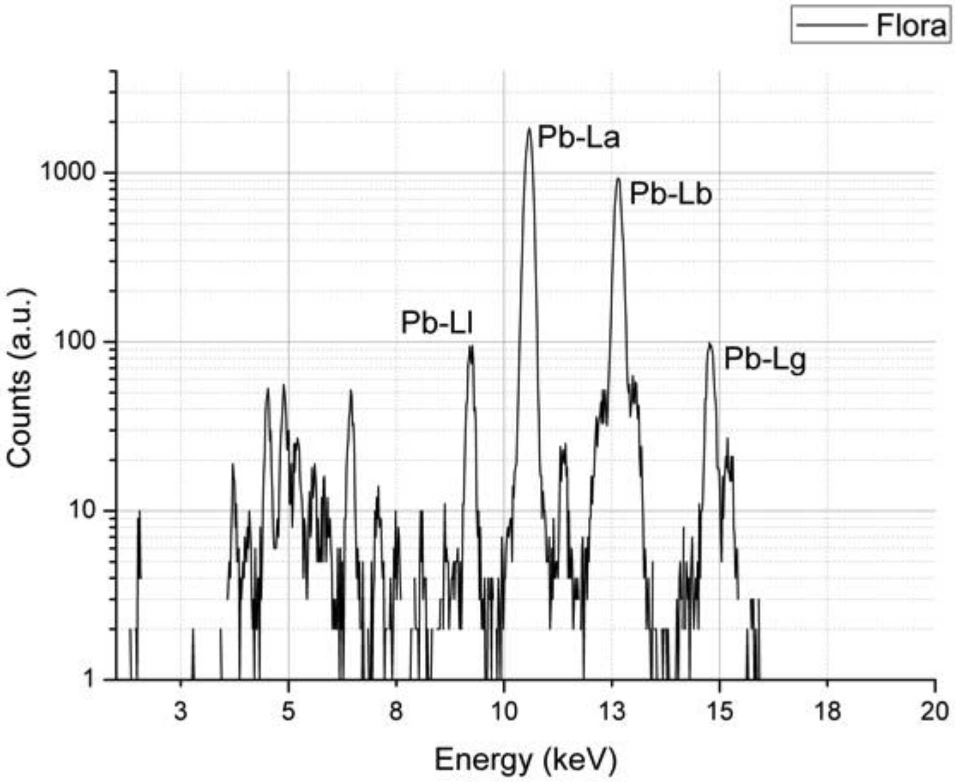

Figure S6. 3 MeV micro- PIXE spectrum measured at NewAGLAE, C2RMF Paris.
